# Elevated temperature drives kelp microbiome dysbiosis, while elevated carbon dioxide induces water microbiome disruption

**DOI:** 10.1371/journal.pone.0192772

**Published:** 2018-02-23

**Authors:** Jeremiah J. Minich, Megan M. Morris, Matt Brown, Michael Doane, Matthew S. Edwards, Todd P. Michael, Elizabeth A. Dinsdale

**Affiliations:** 1 Department of Biology, San Diego State University, San Diego, CA, United States of America; 2 Ibis Biosciences, Carlsbad, CA, United States of America; Universitat Bremen, GERMANY

## Abstract

Global climate change includes rising temperatures and increased *p*CO_2_ concentrations in the ocean, with potential deleterious impacts on marine organisms. In this case study we conducted a four-week climate change incubation experiment, and tested the independent and combined effects of increased temperature and partial pressure of carbon dioxide (*p*CO_2_), on the microbiomes of a foundation species, the giant kelp *Macrocystis pyrifera*, and the surrounding water column. The water and kelp microbiome responded differently to each of the climate stressors. In the water microbiome, each condition caused an increase in a distinct microbial order, whereas the kelp microbiome exhibited a reduction in the dominant kelp-associated order, Alteromondales. The water column microbiomes were most disrupted by elevated *p*CO_2_, with a 7.3 fold increase in Rhizobiales. The kelp microbiome was most influenced by elevated temperature and elevated temperature in combination with elevated *p*CO_2_. Kelp growth was negatively associated with elevated temperature, and the kelp microbiome showed a 5.3 fold increase Flavobacteriales and a 2.2 fold increase alginate degrading enzymes and sulfated polysaccharides. In contrast, kelp growth was positively associated with the combination of high temperature and high pCO2 ‘future conditions’, with a 12.5 fold increase in Planctomycetales and 4.8 fold increase in Rhodobacteriales. Therefore, the water and kelp microbiomes acted as distinct communities, where the kelp was stabilizing the microbiome under changing *p*CO_2_ conditions, but lost control at high temperature. Under future conditions, a new equilibrium between the kelp and the microbiome was potentially reached, where the kelp grew rapidly and the commensal microbes responded to an increase in mucus production.

## Introduction

Kelps form large forests in coastal marine ecosystems that provide habitat for hundreds of species, including benthic and pelagic fishes, invertebrates, vegetation and microbes [1–4]. Microbial communities associated with macroalgae are generally host specific, vary seasonally, and fluctuate based on host condition [5,6]. The host microbiome may represent a majority fraction of the host’s total cellular material and provide the host with additional genomic and metabolic potential [7,8]. Thus, describing and measuring microbial responses to environmental stresses is paramount for evaluating host health.

The marine environment contains over 1800 known species of brown algae, yet the microbial communities or microbiomes have been described in less than 2% [9]. Macroalgal microbiomes have core functions related to vitamin synthesis, biofilm formation, and algae polysaccharide catabolism [9]. Microbiomes benefit the algae by producing micronutrients like B_12_, enhancing iron uptake, and aiding host immune health by producing antibiotics to restrict colonization of pathogenic bacteria [10–12]. Microbes also negatively impact the algae through disease. While several specific pathogens are described in disease of marine organisms, many pathogens remain unknown and disease to the host occurs because of a shift in the types or functions of the microbes present. Dysbiosis, which is a shift in the microbiome from a stable state to disturbed state where commensal or new colonizers may become opportunists pathogens, occurs when the host system is stressed by abiotic or biotic challenges [13,14]. Determining whether the *M*. *pyrifera* microbiome is altered by environmental conditions, such as those associated with global climate change, and whether those alterations affect the function of the host remains an outstanding question.

In the last 150 years global atmospheric concentrations of carbon dioxide have increased 40% to 407 ppm, with an expected increase to 1000 ppm by 2100 leading to a decrease in pH by 0.3–0.4 units [15,16]. Sea surface temperature is predicted to increase by between 1.5 to 4 ^o^C by 2100 [17], while much of the excess carbon dioxide and heat will be absorbed by the ocean. These abiotic fluctuations associated with climate change are expected to be the most extreme in nearshore coastal communities [17], such as those dominated by *M*. *pyrifera*. A recent review of over 100 macroalgae revealed that non-calcifying fleshy macroalgae growth and photosynthesis may increase in an elevated *p*CO_2_ ocean. Most macroalgae, including *M*. *pyrifera*, utilize both CO_2_ and bicarbonate (HCO_3_-) as an inorganic carbon source in photosynthesis and therefore an increase in CO_2_ will reduce use of the less efficient bicarbonate pathway [18]. In contrast, increasing temperature poses risks to macroalgae, especially those living near to their maximum temperature tolerances [19,20]. Enhanced growth and development of the microscopic gametophyte stage of *M*. *pyrifera* occurred with elevated *p*CO_2_ [21,22], yet growth and photosynthesis of the adult macroscopic sporophyte stage are less affected by fluctuations of *p*CO_2_ and more impacted by nutrient availability and elevated temperature [23,24]. *M*. *pyrifera* growth and reproduction declines at 18–20 ^o^C [1,25–27]. Elevated temperature causes reductions in frond elongation rates in kelp [28], while gametophytes have maximal photosynthetic rates occurring between 15–20 ^o^C declining at 30 ^o^C [29]. Temperatures above 18 ^o^C have a negative effect on *M*. *pyrifera* growth, spore production, germination and recruitment [19,30]. In contrast, the combined effects of elevated temperature and *p*CO_2_ together increase *M*. *pyrifera* sporophyte growth and photosynthesis [31], but resulted in higher spore mortality and decreased germination [21]. While these ecological and physiological studies have evaluated kelp in relation to changing abiotic conditions [23,27,31–33], few have investigated the microbes, which are a crucial explanatory variable for describing macroalgal health as measured by growth or decay.

As abiotic conditions vary with climate change, microbial metabolic activity and community dynamics are affected. Under elevated temperature regimes, marine microbial heterotroph production and respiration increase [34], and microbial nitrogen uptake and metabolism intensify [35]. Increases in *p*CO_2_ was positively correlated with higher abundances of *Flavobacteriaceae* and *Rhodobacteraceae* bacteria in sediments, which contribute to higher organic decomposition and a lower abundance of nitrifying bacteria [36]. In a mesocosm experiment where transplanted kelps were grown under elevated *p*CO_2_ conditions, shifts occurred in water column microbial community structure, but the kelp microbiome was not assessed. Increases in the relative abundance of Gammaproteobacteria and genes relating to iron acquisition and membrane transport, and decreases in the relative abundance of Flavobacteria were associated with increased *p*CO_2_ [37]. While we have shown that microbes in the water column surrounding kelp are affected by changes in *p*CO_2_, the effects on the microbial community growing on the kelp surface requires evaluation.

The purpose of our case study was to evaluate the individual and combined effects of elevated temperature and *p*CO_2_ associated with climate change on the physiology of the giant kelp, *M*. *pyrifera*, its microbiome, and the microbes in the surrounding water column. We hypothesized that the abiotic stressors may affect the microbiomes associated with the kelp and potentially interrupt some of the normal microbiome functions. We demonstrate that the microbiomes of the kelp and water are uniquely affected by changes in *p*CO_2_ and temperature, and propose mechanistic links between the microbiome and kelp growth or decay in the context of climate change.

## Results

**Fig 1:**
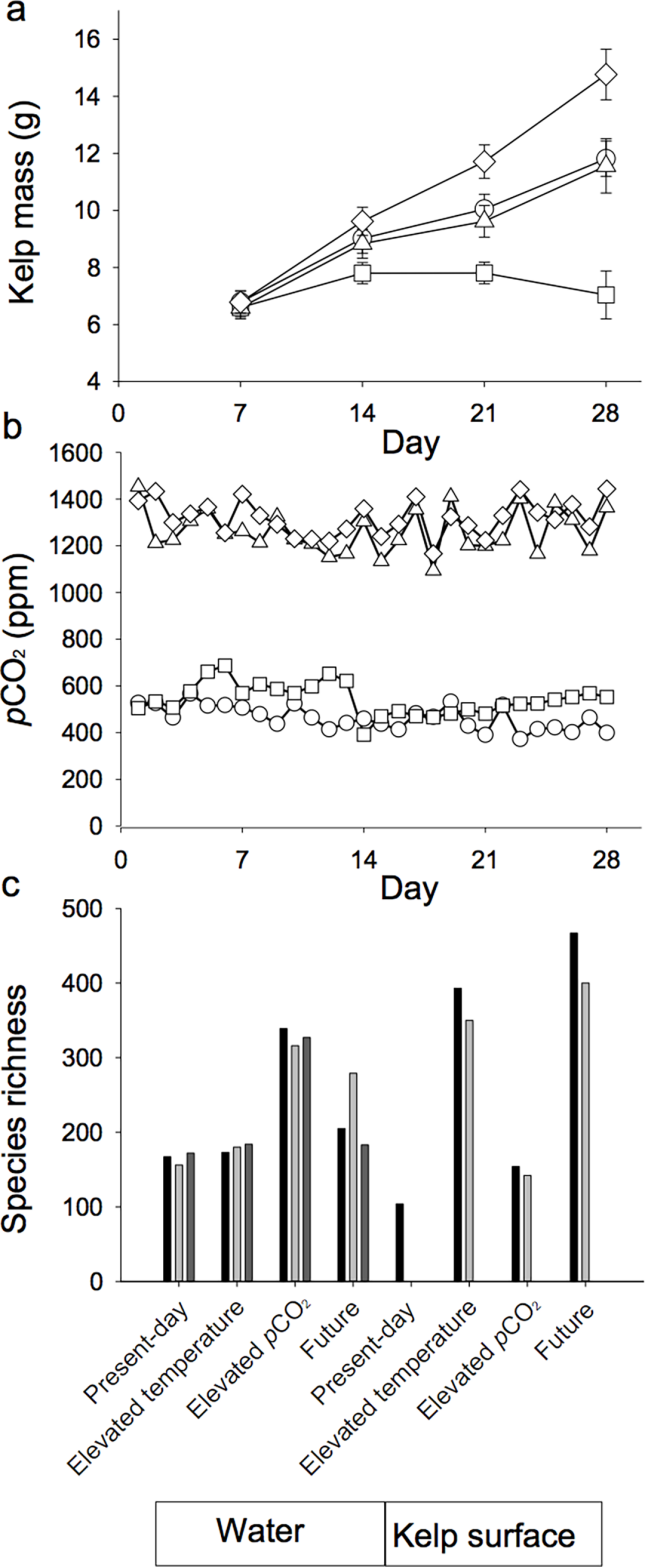
Experimental conditions of kelp mesocosm. Kelp was grown for four weeks in four different abiotic stressors, present-day 12 ^o^C 500 ppm of CO_2_ ○, Elevated Temperature 15 ^o^C 500 ppm of CO_2_ □, Elevated *p*CO_2_ 12 ^o^C 1300 ppm of CO_2_ △, and future conditions 15 ^o^C 1300 ppm of CO_2_ ◇. (a) Growth of kelp over the duration of the experiment. Each week, 18 fronds were sampled and weighed per incubation mesocosm. (b) Elevated *p*CO_2_ was maintained between 1,200 and 1,400 ppm of CO_2_ whereas temperature was held constant at either 12 ^o^C or 15 ^o^C. (c) microbiome species richness. At week 4, microbial communities were sampled from the water and kelp surfaces and sequenced. Alpha diversity, Shannon species richness, was calculated for all microbiome samples using MG-RAST (grey scale represents replicates; three for water and 2 or 1 for kelp surface).

Kelp growth and photosynthetic Carbon uptake was reduced in the elevated temperature treatment, but not affected by increased *p*CO_2_, and significantly increased in future conditions (Fig 1A). A total of 19 of the possible 24 microbiomes were generated using whole genome shotgun sequencing from the 12 water column samples (3 replicates per treatment mesocosm) and seven kelp surface samples. Out of the 12 kelp surface samples (3 replicates per treatment mesocosm), only three original samples had at least 100 ng of DNA to make libraries. For the samples with less than 100 ng of DNA, replicates were pooled yielding a total of 7 metagenomic libraries. The microbiomes comprised 14.9 Gb of data, with over 45 million reads and an average of 2.4 million reads per treatment (S1 Table). Over 18 million known proteins were annotated at approximately one million proteins per microbiome. Across the 19 microbiomes, a total of 2,693 genera and 1,097 gene function categories were annotated. Applying the lower cutoff (S1 Fig), the genera were reduced to a total of 361, including 329 genera shared in the water column and kelp surface, consisting of a mean loss of 1.3 ± 0.1% sequences per microbiome. The gene function list was reduced from 1,097 to 718 gene functions, with 689 shared between the water column and kelp surface, consisting of a loss of 3.1 ± 0.178% of sequences per microbiome.

### Microbial community analysis

Rarefaction curves indicate microbiomes were sequenced at a depth sufficient to evaluate taxonomical and functional features (S2 Fig). For alpha diversity, water column microbial richness ranged from 165 to 327 species, whereas the kelp surface microbial richness ranged from 104 to 433 species (Fig 1). In the water column microbiomes, the elevated *p*CO_2_ mesocosm had a higher species richness of 327 ± 6.6, compared to present day (165 ± 4.7), elevated temperature (179 ± 3.2), and future conditions (222 ± 29.0) (F_df = 3_ = 23.46, P ≤ 0.01), (Tukey HS, P ≤ 0.01). In the kelp surface microbiomes, elevated temperature and future conditions had a species richness of 371 ± 21.5 and 433 ± 33.5 and present day microbiomes had 104 species and elevated *p*CO_2_ had 148 ± 6.0 species (Fig 1).

#### Water and kelp surface microbiomes were impacted differently by global climate stressors

**Fig 2:**
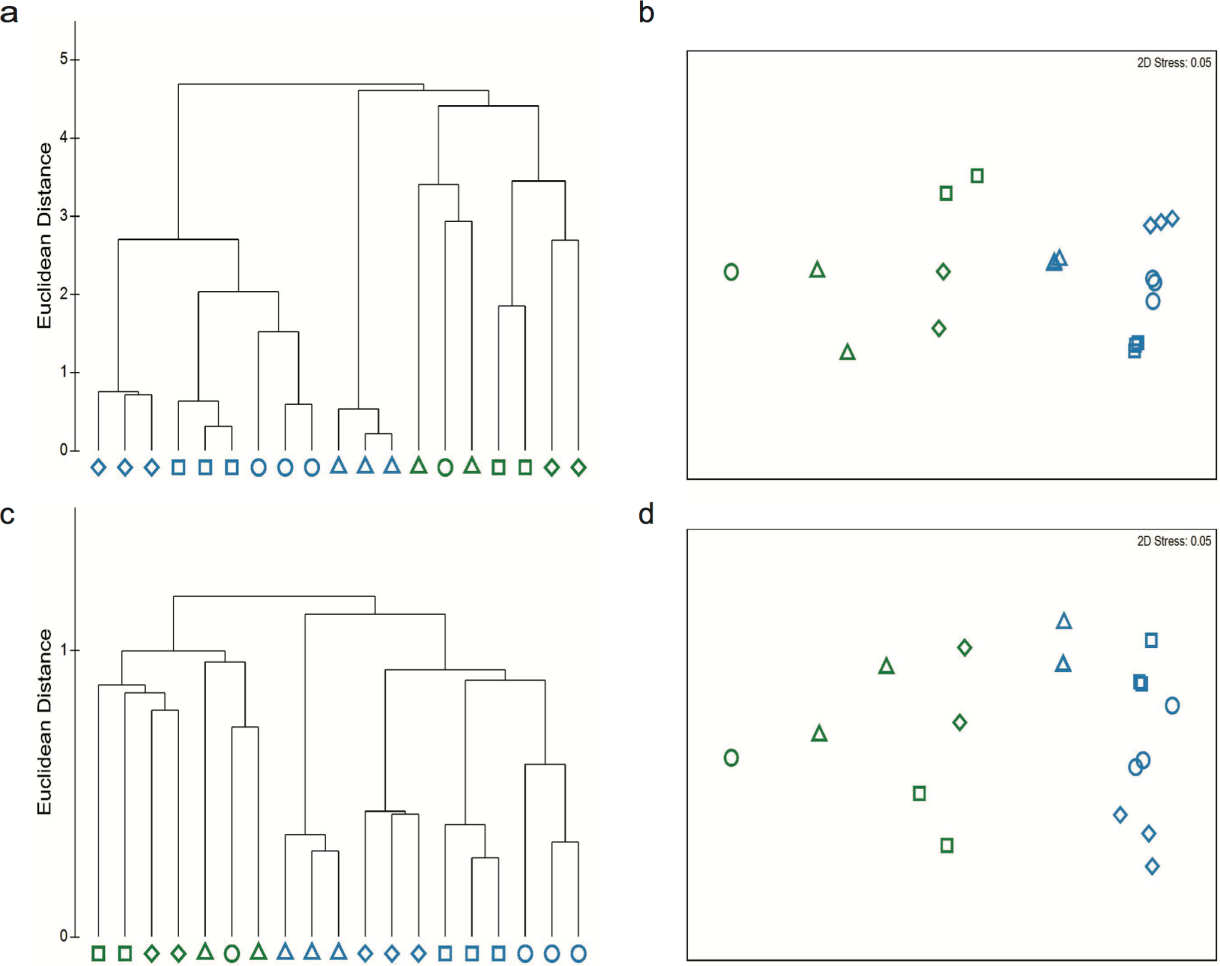
Microbial community analysis of water column and kelp surfaces across treatments. The relatedness of the microbiome from the water (blue) and kelp surface (green) calculated using hierarchical clustering, and nMDS ordination (present-day ○, elevated temperature □, elevated *p*CO_2_ △, and future ◇) for (a) genera cluster analysis, (b) Genera nMDS plot, (c) SEED gene function level 3 cluster analysis, (d) SEED Gene Function level 3 nMDS plot.

Based on taxonomical composition, the microbiome of the water column was significantly different from the microbiome from the kelp surfaces and microbiomes from each mesocosm clustered (PERMANOVA Pseudo-F = 118.21, P ≤ 0.001) (Table 1A, Fig 2) and explained the largest effect (49.0%) at the genera level. Climate stressors affected each of the microbial communities (Pseudo-F = 16.31, P ≤ 0.001) with a magnitude of effect size of 12.0%, while there was also an interaction between the effect of the climate treatment and microbiome environment (Pseudo-F = 21.65, P ≤ 0.001), magnitude of effect size of 35.0%. This large interaction effect indicates climate stressors altered the water column and kelp surface microbiomes uniquely. The increase in *p*CO_2_ had the largest effect on the taxonomic components of water column (23.7% dissimilarity), whereas elevated temperature had the highest effect on the kelp microbiome (51.8% dissimilarity) (Table 1B, Fig 2).

**Table 1:** (a) PERMANOVA and (b) SIMPER results indicate microbial communities differ taxonomically and functionally between environment (water and kelp surface) and across climate change related stressors.

a. PERMANOVA	df	Sum of squares	Mean of squares	Pseudo-F	*P* (perm)	Mag. of effects %
Genera						
Environment	1	157.73	157.73	118.21	< 0.001	49
Climate change	3	65.30	21.77	16.31	< 0.001	12
Interaction	3	86.66	28.89	21.65	< 0.001	35
Residual	11	14.68	1.33			4
Total	18	324.36				100
Gene Function						
Environment	1	10.91	10.91	62.29	< 0.001	47
Climate change	3	5.19	1.73	9.87	< 0.001	13
Interaction	3	5.96	1.99	11.33	< 0.001	33
Residual	11	1.93	0.18			7
Total	18	23.98				100
b. SIMPER pairwise analyses					% Dissimilarity	
Genera	Environment	Water vs. Kelp surface			54.4	
Water	Climate change	present-day vs. + temperature			8.5	
		present-day vs. + *p*CO2			23.7	
		present-day vs future			10.0	
Kelp surface	Climate change	present-day vs. + temperature			51.8	
		present-day vs. + *p*CO2			17.0	
		present-day vs future			46.4	
Gene Function	Environment				3.9	
Water	Climate change	present-day vs. + temperature			1.1	
		present-day vs. + *p*CO2			1.9	
		present-day vs future			1.3	
Kelp surface	Climate change	present-day vs. + temperature			3.3	
		present-day vs. + *p*CO2			1.3	
		present-day vs future			3.7	

The distribution of microbial functions across microbiomes followed a similar pattern as the genera analysis with water and kelp microbiomes forming separate clusters (Fig 2). Based on the gene function composition, there was a significant difference between the microbiomes in the water column and kelp surface (Pseudo-F = 62.29, P ≤ 0.001) (Table 1A), with these differences in environments explaining 47.0% of the variation among treatments in the statistical model. Temperature and *p*CO_2_ also affected the functional profile of the microbiomes (Pseudo-F = 9.87, P ≤ 0.001), but this was not consistent in the two environments (environment x climate stressor interaction Pseudo-F = 11.33, P ≤ 0.001) (Fig 2C) (Table 1B). The interaction between environment and climate stressors had the second biggest effect in the model (33%). Elevated *p*CO_2_ treatment induced the greatest change in the water column microbiome, as the functional profiles showed 1.9% dissimilarity compared with the present day microbiome. The functional profile of the present day kelp surface microbiome was most dissimilar to the profile of the future and elevated temperature treatments (Table 1B).

#### Water column and kelp surface have unique microbiomes

**Fig 3:**
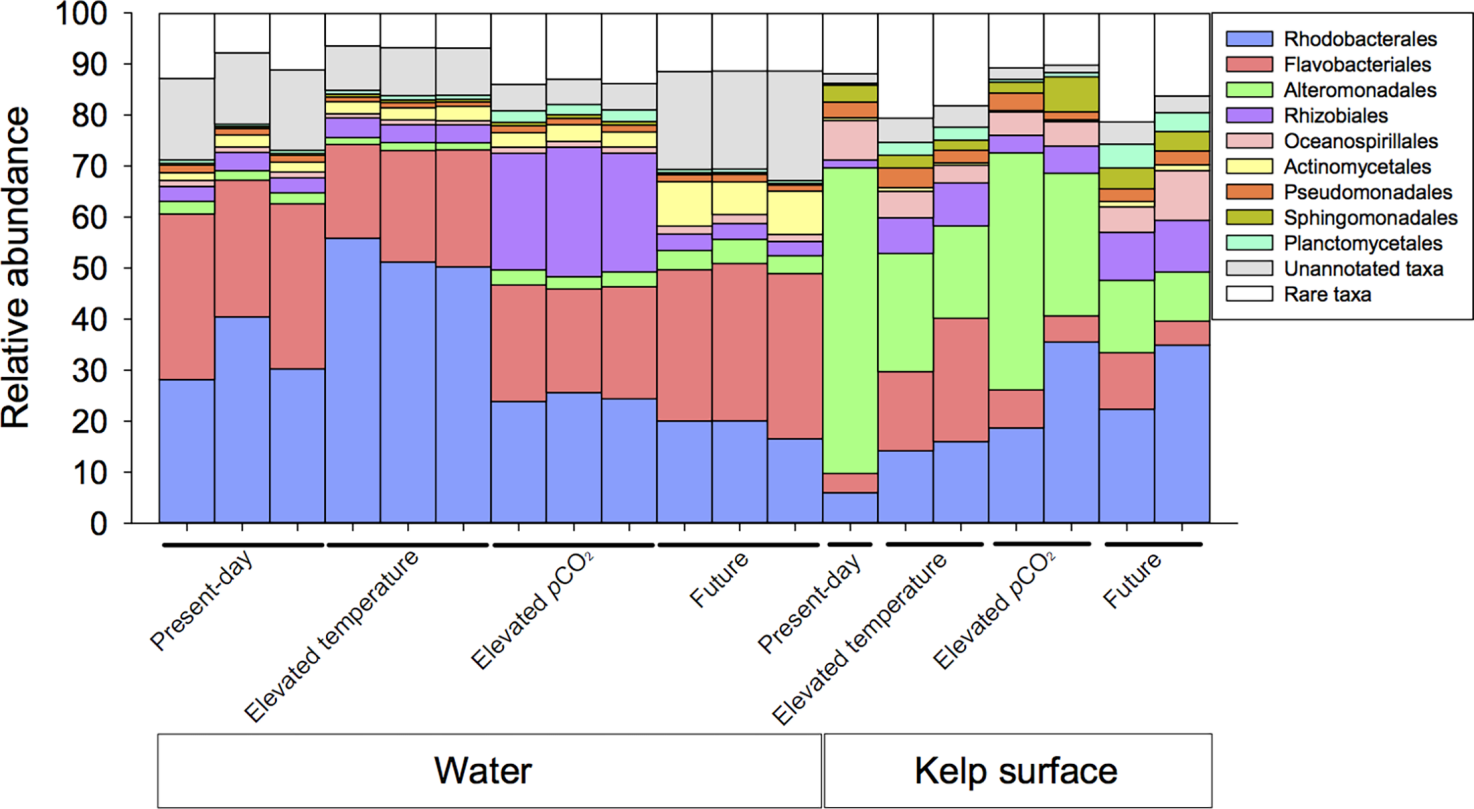
Taxonomic differences in community composition of water and kelp surface microbiomes. Taxonomic compositions of the ten most abundant bacteria orders, in the water column and kelp surface microbiomes from present-day, elevated temperature, elevated *p*CO_2_, and future conditions. Rare taxa as labeled in the legend, are microbial orders not in the top ten.

**Fig 4:**
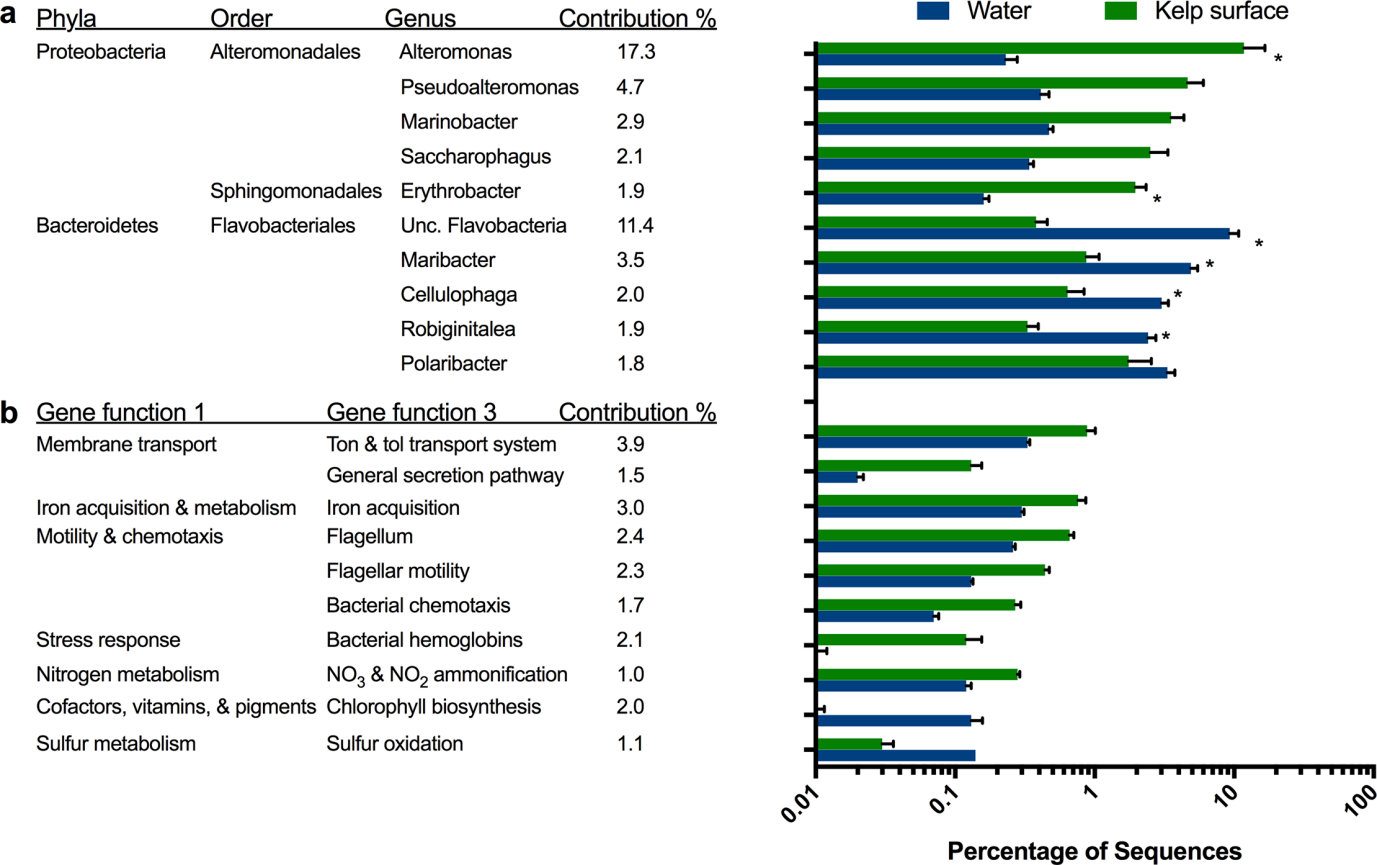
Strongest drivers of microbiome compostional changes in the water and kelp. The top ten bacterial genera (a) and gene functions (b) differentiating the water column from the kelp surface microbiomes as determined by SIMPER analysis.

Across all treatment groups, the water and kelp microbiomes were distinctive, as were the communities’ response to climate stressors (Fig 3). In all four treatments, the water microbiomes had higher proportion of Flavobacteriales (26.1% ± 2.7 mean and s.e. percent composition in the microbiome) and Actinomycetales (3.8% ± 1.4), while the kelp surface microbiome was enriched with Alteromonadales (35.4% ± 10.6), Oceanospirillales (6.0% ± 0.9) and Sphingomonadales (3.5% ± 0.5) (Fig 3). Similar differences in taxa between the water column and kelp surface occur in Pt Loma kelp forest (S3 and S4 Figs). The Alteromonadales order accounted for 26.9% of the observed variation between the water and kelp surface microbiomes and the genera *Alteromonas*, *Pseudoalteromonas*, *Marinobacter* and *Saccharophagus* genera were enriched by an average of 52.2, 11.3, 7.6, and 7.3 fold, respectively, on the kelp surface compared with the water column. The *Erythrobacter* genus within the Sphingomonadales order was enriched 11.9 fold on the kelp surface (Fig 4A). The water microbiome was enriched with the Flavobacteriales order and had an increased composition of *Maribacter*, *Cellulophaga*, *Robiginitalea*, and *Gramella* genera at 5.8 fold, 4.9 fold, 7.6 fold, and 3.2 fold, respectively (Fig 4A). The Rhodobacteriadales order was also over represented in the water microbiome (32.2% ± 7.3) compared to the kelp microbiome (19.22% ± 5.35).

Several functions were enriched in the kelp surface microbiome compared to the water microbiomes (Fig 4B). The kelp microbiomes were enriched with genes related to membrane transport, iron acquisition and metabolism, motility and chemotaxis, stress response, and nitrogen metabolism. Within the membrane transport system, the Ton and Tol transport system and general secretion pathways were enriched 2.7 and 8.0 fold on the kelp surface, while iron acquisition was increased 2.5 fold. Within the motility and chemotaxis category; flagellum, flagellar motility and bacteria chemotaxis genes were enriched in the kelp surface microbiomes, 2.5, 3.3, and 3.7 fold, respectively. Three bacterial orders: Rhodobacterales, Flavobacterales, and Alteromonadales, within the water column and kelp surface contained the most flagellar motility genes although the distributions were unique to the water column and kelp surface. Specifically, the water column was mostly comprised of Rhodobacterales (W: 36%; KS: 17%) and Flavobacterales (W: 29%; KS: 1%) while Alteromonadales dominated the kelp surface (W: 10%; KS: 50%). Among the virulence, antibiotics, and toxins category, genes related to cobalt-zinc-cadmium resistance and multidrug resistance efflux pumps were enriched by 1.4 and 1.8 fold respectively on the kelp surface compared with the water microbiome. In both water and kelp surface microbiomes, the Acriflavin resistance protein comprised 63–86% of multidrug efflux pumps. Under nitrogen metabolism, nitrate and nitrite ammonification genes were enriched by 2.4 fold in the kelp surface microbiome and were primarily Nitrite reductase NADPH large subunit EC 1.7.1.4 (mean 20.7%), assimilatory nitrate reductase large subunit EC 1.7.99.4 (mean 19.1%), and the Nitrate ABC transporter, nitrate-binding protein (mean 13.7%). The water microbiome was enriched in cofactors, vitamins, prosthetics group, and sulfur metabolism. Chlorophyll biosynthesis and sulfur oxidation were enriched by 15.3 and 4.4 fold, respectively, in the water column microbiome. The Chlorophyll biosynthesis group had many genes but Protoporphyrin IX Mg-chelatase subunit H EC 6.6.1.1 and Chlorophyllide reductase subunit BchZ EC 1.18 were the two most dominant comprising on average 20% and 8% respectively.

#### Water microbiomes were most influenced by elevated pCO_2_

**Fig 5:**
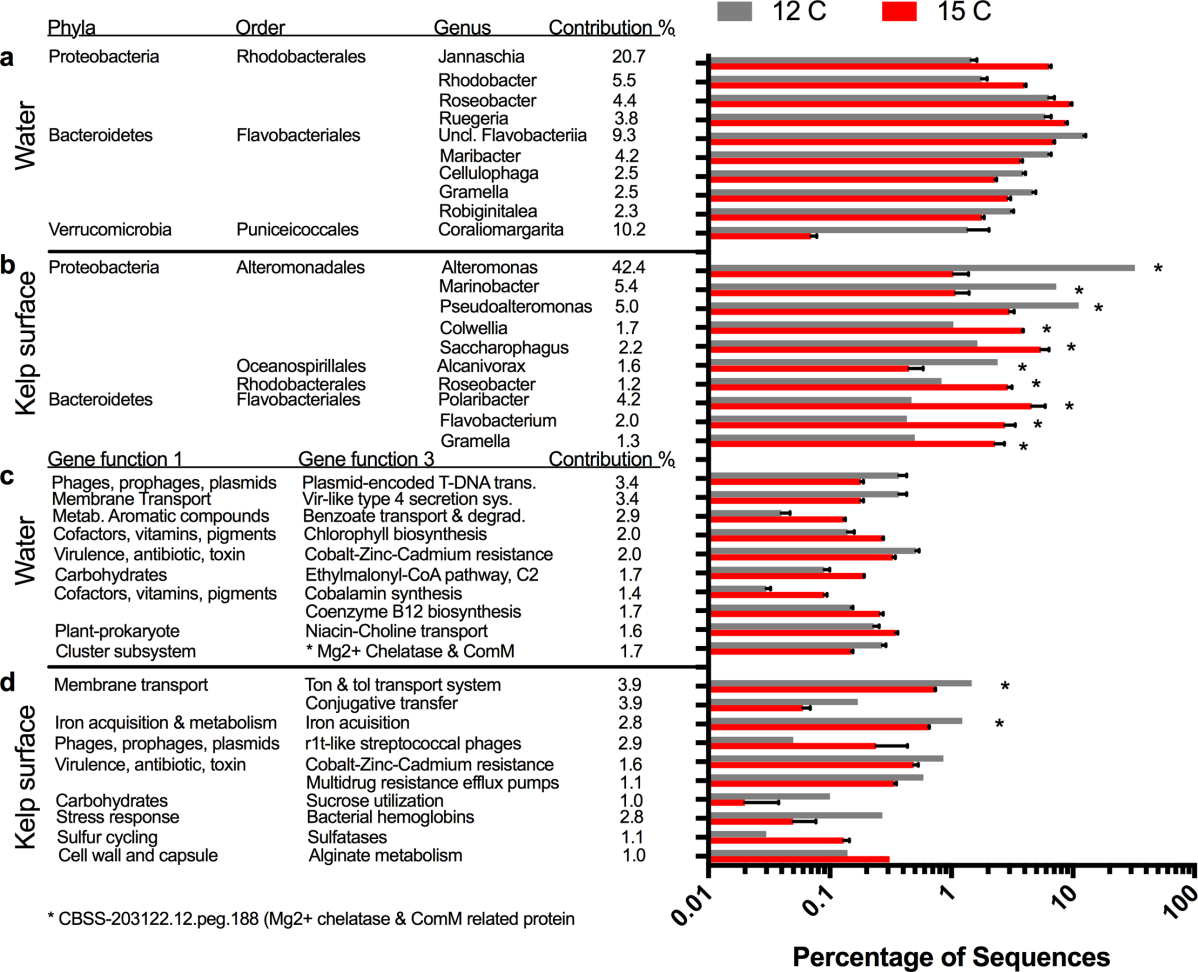
Effects of temperature on water and kelp surface microbiomes. Top ten microbial genera and gene functions from the water and kelp surface environments driving the differences between present-day and elevated temperature. Microbial genera in the water column (a) and kelp surface (b) at elevated temperatures were 8.5% and 51.8% dissimilar compared to present day conditions by SIMPER analysis. Microbial gene functions in the water column (c) and kelp surface (d) at elevated temperatures were 1.1% and 3.3% dissimilar compared to present-day conditions.

**Fig 6:**
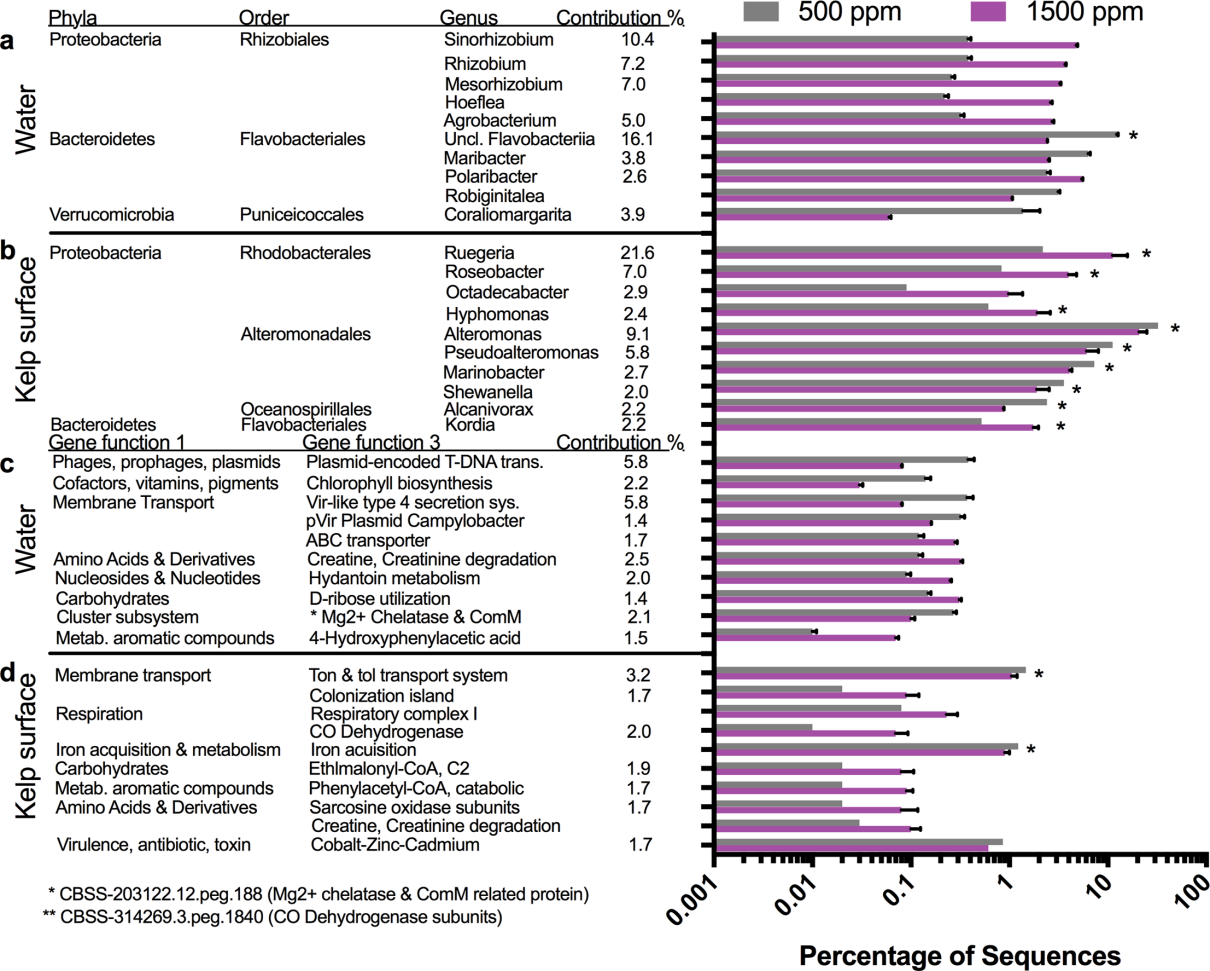
Effects of pCO_2_ on water and kelp surface microbiomes. Top ten microbial genera and gene functions from the water and kelp surface environments driving the differences between present-day and elevated pCO_2_. Microbial genera in the water column (a) and kelp surface (b) at elevated pCO_2_ were 23.7% and 17.0% dissimilar compared to present day conditions by SIMPER analysis. Microbial gene functions in the water column (c) and kelp surface (d) at elevated pCO2 were 1.9% and 1.3% dissimilar compared to present-day conditions.

**Fig 7:**
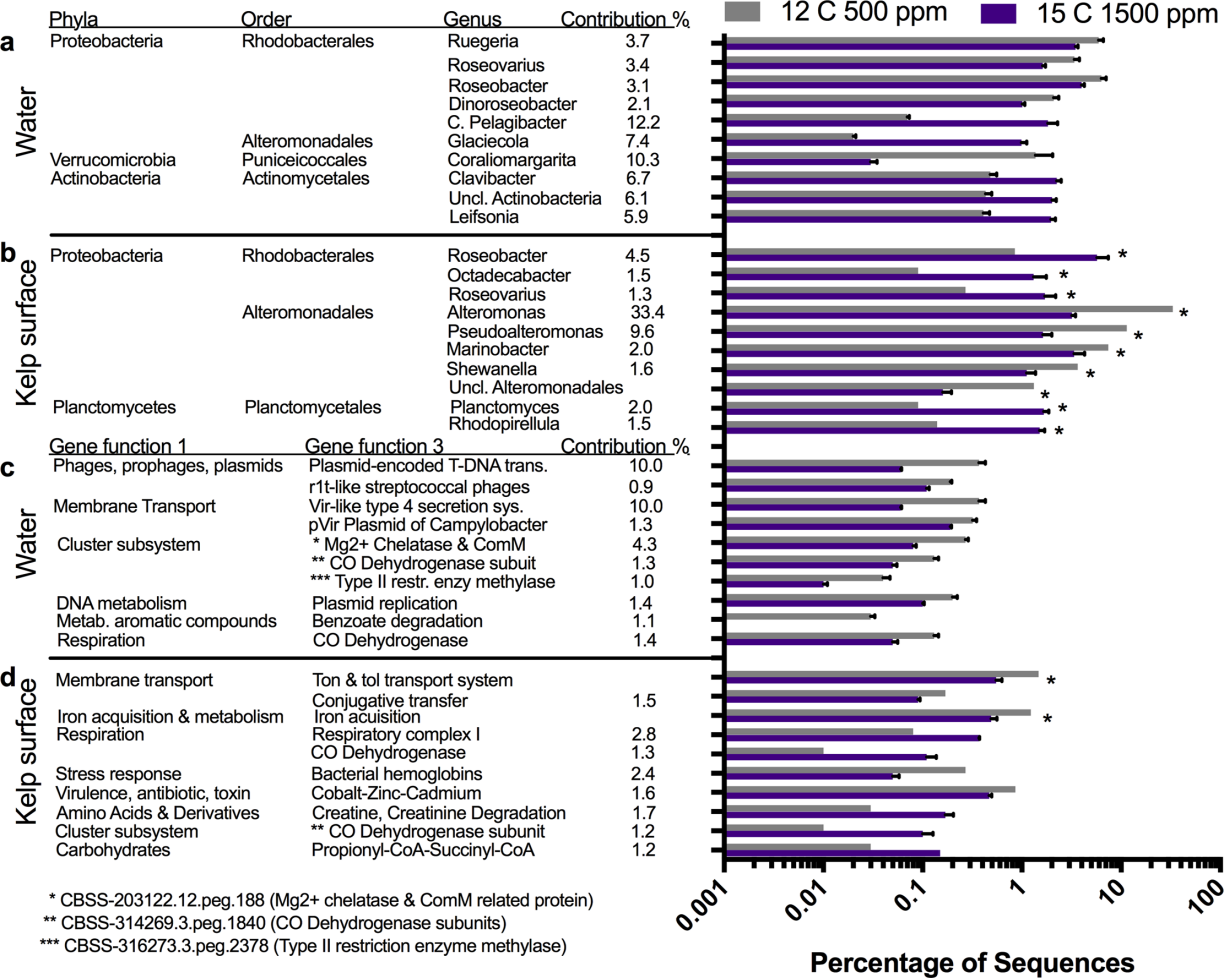
Combined effects of temperature and pCO_2_ on water and kelp surface microbiomes. Top ten microbial genera and gene functions from the water and kelp surface environments driving the differences between present-day and future. Microbial genera in the water column (a) and kelp surface (b) at future were 10.0% and 46.4% dissimilar compared to present day conditions by SIMPER analysis. Microbial gene functions in the water column (c) and kelp surface (d) at elevated pCO2 were 1.3% and 3.7% dissimilar compared to present-day conditions.

Microbial orders in the water microbiome were differentially affected by changes in environmental conditions (Fig 3) and each condition had a single order that increased in abundance from the present day. Elevated *p*CO_2_ showed an increase in the order Rhizobiales, elevated temperature showed an increase in Rhodobacterales, and the combination of *p*CO_2_ and temperature showed an increase in Actinomycetales. These changes were also reflected in alterations at the genus level for the various conditions of elevated temperature (Fig 5), elevated pCO2 (Fig 6), and combined elevated temperature and pCO2 (Fig 6). The elevated *p*CO_2_ caused the largest effect on the microbiome composition and was 23.7% dissimilar compared with the present day microbiome, whereas elevated temperature was 8.5% dissimilar and future conditions were 10.0% from the present day microbiome (Table 1B). During elevated *p*CO_2_, seven genera within the order Rhizobiales increased in abundance including *Sinorhizobium* (12.6 fold increase), *Rhizobium* (9.7 fold), *Mesorhizobium* (12.5 fold), *Hoeflea* (11.9 fold), and *Agrobacterium* (8.5 fold) (Fig 6A), whereas the abundances of several genera within the phyla Bacteroidetes and Verrucomicrobia decreased (Fig 6A). Elevated temperature was associated with an increase in the proportion of Rhodobacterales order in the water microbiome, (Fig 3) including increases in the genera *Jannaschia* (4.4 fold), *Rhodobacter* (2.3 fold), *Roseobacter* (1.5 fold), and *Ruegeria* (1.5 fold) (Fig 5A). Elevated temperature also reduced the proportion of Bacteroidetes and Verrucomicrobia in the water microbiome. The combination of elevated *p*CO_2_ and temperature was associated with an increase in the proportion of Actinomycetelales in the water microbiome and a reduction in the proportion of Rhodobacterales. Various genera within this order increased in abundance including: *Clavibacter* (4.6 fold), unclassified Actinobacteria (4.7 fold) and *Leifsonia* (4.7 fold). Further, a 49.6 fold increase in the *Glaciecola* genus within the Alteromonadales order and a 43.4 fold decrease in the *Coraliomargarita* genus within the Puniceicoccales order occurred in the future mesocosm (Fig 7A).

When comparing water communities based on the gene function composition, elevated *p*CO_2_ was the most dissimilar from the present-day at 1.9% dissimilar, followed by future at 1.3% and elevated temperature at 1.1% (Table 1B). The gene functions driving the differences in elevated temperature, elevated *p*CO_2_, and future conditions were related to a variety of categories including membrane transport, carbohydrate metabolism, virulence factors and plasmids (Fig 5C, Fig 6C, Fig 7C).

#### Kelp surface microbiomes were most influenced by elevated temperature and future conditions

Microbial orders in the kelp microbiome were differentially affected by changes in environmental conditions (Fig 3), while each condition showed a general decline in Alteromonadales, increase in Flavobacteriales, Rhodobacterales and Rhizobiales compared to the present day microbiome. The elevated temperature had the largest effect on the kelp microbiome composition, being 51.8% dissimilar compared with the present day microbiome, whereas elevated *p*CO_2_ was 17.0% dissimilar, and future conditions were 46.4% dissimilar from the present day microbiome (Table 1B). In elevated temperature, three genera within the order Alteromonadales declined in abundance, *Alteromonas* (-31.0 fold), *Marinobacter* (-6.7 fold) and *Pseudoaltermonas* (-3.7 fold), and two genera, *Colwellia* (3.7 fold) and *Saccharophagus* (3.3 fold) increased in abundance. The order Flavobacteriales increased in abundance with increased temperature, including the genera *Polaribacter* (9.9 fold), *Flavobacterium* (6.4 fold) and *Gramella* (4.6 fold) (Fig 5B). Elevated *p*CO_2_ caused genera within the order Alteromonadales to decline in abundance, *Alteromonas* (-1.6 fold), *Marinobacter* (-1.8 fold) and *Pseudoaltermonas* (-1.8 fold), and *Shewanella* (-1.9 fold), but the proportional decline was slight compared with the temperature treatment. The proportion of Rhodobacterales order increased (Fig 3), including increases in the genera *Ruegeria* (5.1 fold), *Roseobacter* (4.8 fold), *Octadecabacter* (11.5 fold) and *Hyphomonas* (3.1 fold) (Fig 6B). The combination of elevated *p*CO_2_ and temperature caused a major decline in the order Alteromonadales, including the genera *Alteromonas* (-10.3 fold), *Pseudoaltermonas* (-7.0 fold), *Marinobacter* (-2.2 fold) and *Shewanella* (-3.3 fold). In addition, the proportion of Rhodobacterales (27.7 fold), and Planctomycetales increased 29.2 fold in abundance in the future kelp microbiomes (Fig 7B).

The evaluation of the functional profiles of the kelp microbiome showed that the future conditions were most dissimilar to the present day conditions (3.7% dissimilar), followed by elevated temperature (3.3%) and elevated *p*CO_2_ (1.3%) (Table 1B). The gene functions driving the differences in the elevated temperature regime were a 2.0 fold decrease in Ton and Tol transport systems, 1.9 fold decrease in iron acquisition, and 4.9 fold increase in r1t-like streptococcal phages, 2.2 fold increase in alginate metabolism, and 4.4 fold increase in sulfatases (Fig 5D). For elevated *p*CO_2_, Ton and Tol transport systems and iron acquisition were reduced, while a 2.8 fold and 6.0 fold increase in respiratory complex I and CO dehydrogenase contributed to functional differences (Fig 6D). The enrichment in CO dehydrogenase genes associated with increased CO_2_ on the kelp surface (Figs 6D and 7D), primarily belonged to the Ruegeria and Roseobacter genera which were also elevated at the taxonomic level (Figs 6B and 7B). In the future conditions incubation mesocosms, Ton and Tol transport systems and iron acquisition were both reduced 2.7 and 2.5 fold while respiratory complex I and CO dehydrogenase were enriched 4.4 and 8.8 fold (Fig 7D). The Alteromonadales order contains the majority of the genes (mean 57%) annotated to Ton and Tol transport system with the TonB-dependent receptor being the most prominent gene (mean 50%). Alginate metabolism genes included alginate lyases (mean 47%), phosphomannomutase (mean 16%), mannose-6-phosphate isomerase (7%), poly(beta-D-mannuronate) lyase (15%), and poly(beta-D-mannuronate) O-acetylase (8%) suggesting the primary polysaccharides being alginate and mannose. During elevated temperature, the alginate lyase genes were enriched compared to all other metagenomes (mean 63% vs. 40%) while the phosphomannomutase genes were lower (mean 9% vs. 19%). Furthermore, the bacteria associated with alginate lyase genes on the kelp surface were primarly *Pseudoalteromonas* (mean 23%) and *Saccharophagus* (mean 26%) with *Saccharophagus* becoming enriched in the elevated temperature to (38%).

## Discussion

Macroalgae development, growth and decay are facilitated by bacteria and bacterial physiology is tied to surrounding abiotic conditions, such as temperature, pH and nutrient availability. Our case study experiment tested the independent and combined effects of elevated temperature and *p*CO_2_ on the microbiomes of the *M*. *pyrifera* kelp surface and surrounding water column. Microbiomes of the water column and kelp surface were unique, and were affected differently by global climate change stressors. The water column microbiome was altered most by increased *p*CO_2,_ while the kelp surface microbiome was altered most by increased temperature and future conditions. The disturbances of the kelp microbiome in elevated temperature correlated with decreased kelp growth, while the future condition microbiome associated with prolific kelp growth. Combined effects of future conditions were significant on the kelp surface microbiome indicating the importance of multifactorial experimental designs addressing multiple variables to evaluate ecological consequences of climate change stressors on host organisms. Although our overall replication of study conditions were limited due to technical constraints, our intra-tank replicates were consistent and provide a foundation for future targeted studies both *in situ* and in the lab.

### Climate stressors induced changes to water microbiomes

The water microbiome showed a distinct increase in single bacterial orders with each climate change condition with elevated *p*CO_2_ being the most influential. These microbiomes had the highest alpha diversity and a 7.3 fold increase in Rhizobiales. The Rhizobiales order assimilates dissolved inorganic carbon (e.g. CO_2_) in coastal environments [38] and could be utilizing excess *p*CO_2_ alongside kelp. In addition, species within the Rhizobiales order have important roles in both denitrification [39] along with nitrogen fixation in soil, and are attached to organic particles in the deep ocean and degrade xenobiotic and refractory compound [40,41]. The functional analysis identified an increase in the proportion of sequences similar to denitrification and 4-hydroxphenylacetic acid catabolic pathways which can stimulate algal growth [42]. An increase in denitrification may be a response to the increase in some Rhizobiales and nitrogen fixation. Nitrogen fixation on *M*. *pyrifera* surfaces is up to ten times higher than other macroalgae and is associated with decomposition [43]. An alternative hypothesis to increased denitrification would be explained by an increase in overall NO_3_ and NO_2_ availability in the ecosystem which also could be a result of bacterial metabolism.

### *M*. *pyrifera* microbiome was dominated by *Alteromonadales* and unique functions

The Alteromonadales order was a major component of the *M*. *pyrifera* microbial community, similar to *in situ* sampling of kelp at Pt Loma (S3 and S4 Figs), and declined in abundance in the altered climate condition treatments. Alteromonadales, while important in the Point Loma kelp forest, were not described in the *M*. *pyrifera* microbiome from Monterey Bay [44] indicating that the frond surface microbiome may vary regionally or alternatively may be attributed to the difference in metagenomic sampling and sequencing methods. Within the Alteromonadales order, *Alteromonas*, *Cytophaga*, *Pseudomonas* and *Pseudoalteromonas* genera contain enzymes like agarases, alginate lyases, cellulases, pectinases, and fucoidanases which catabolize kelp polysaccharides [45] with some causing disease in other species of brown algae [46,47]. However, in this study *Alteromonadales* were enriched on the kelp surfaces during the present day treatment where the kelp was thriving, and was reduced by 51.0% in the elevated temperature treatment where the kelp showed reduced growth. Future work should focus on the strain ecology and metabolic functions of Alteromonadales microbes throughout the life history, geographic range, and seasonality of *M*. *pyrifera*. The kelp surface microbiome was enriched with genes related to nutrient acquisition, motility, and nitrogen metabolism. Genes related to Ton and Tol transport systems, which are important for gram negative marine bacteria to obtain extracellular dissolved organic matter, vitamins and iron from the environment [48,49]. Nitrate and nitrite ammonification related genes were enriched on the kelp surface microbiome suggesting a possible mutualistic mechanism for nitrogen cycling between kelp and bacteria. Other microbes on the kelp surface had an enrichment of genes related to environmental sensing and motility, including bacterial chemotaxis and flagellum, which may be associated with biofilm formation [50] or macroalgae colonization [51]. Furthermore, although we were able to identify candidate gene families associated with algae degradation across all treatments, future studies should strive to link transcriptional or protein profiles of the bacteria to demonstrate functional activity.

### Increased temperature and an altered microbiome reduced kelp growth

Host macroalgae susceptibility to bacterial pathogenesis is increased during elevated temperature regimes and is related to reduced chemical defense responses [5,19]. We found that elevated temperature was associated with a unique shift in the kelp surface microbiome which corresponded with reduced *M*. *pyrifera* growth.The kelp surface microbiome showed a loss of Alteromondales and increase in Flavobacterales indicating that Flavobacterales may be negatively associated with kelp growth. Flavobacteriales strains, including *Flavobacterium sp*. LAD-1 [52], *Kordia algicida* OT-1 [53], *Gramella forsetii* KT0803 [54], and *Cellulophaga lytica* DMS 7489 [55] are algicidal bacteria which contribute to disease in red and brown macroalgae. Similarly, our study identified *Polaribacter*, *Flavobacterium*, and *Gramella* enriched on the kelp surface in the elevated temperature condition and these genera have alginate lyase enzymes which assist in degrading brown algae [55–58]. The functional analysis corroborated the taxonomic change with an increase in alginate metabolism and sulfatases. An increase in *Colwellia* and *Saccharophagus* genera, which contain enzymes that degrade algae polysaccharides [55,59] occurred at higher temperature. *Saccharophagus degradans* 2–40, for example, has numerous enzymes that degrade marine plant cellulose [60] and five agarase enzymes [61]. Similarly in the brown macroalgae, *Fucus vesiculosus*, increased temperature was associated with an increase in Flavobacteriales and reduced kelp growth, while CO_2_ had no effect [62], suggesting a common mechanism across brown macroalgae.

### Kelp grew prolifically in future conditions with marked changes in the microbiome

Elevated temperature combined with elevated *p*CO_2_ were surprisingly associated with increased *M*. *pyrifera* growth [23] and were related to increased microbial diversity. Microbes within the Planctomycetales and Rhodobacterales orders were both enriched on the kelp surface, similar to other studies of microbes associated with macroalgae (Lachnit et al., 2011; Friedrich 2012). Planctomycetales, produce sulfatases enabling catabolism of sulfated polysaccharides and are associated with a variety of hosts, including, marine sponges, corals, ascidians, and macroalgae biofilms [63–65]. Sulfatase genes were increased in the kelp microbiome under future conditions and were primarily comprised of aryl sulfatase, choline-sulfatase, sulfatase, and sulfatase modifying factor 1 precursor all of which were present in the Planctomycetales microbes. Two Planctomycetales genera, *Planctomyces* and *Rhodopirellula*, enriched on the kelp surface, are macroalgae symbionts with resistance to antimicrobial compounds produced by macroalgae [66]. A strain *Rhodopirellula baltica SH1*, contains 110 different sulfatase genes [64]. Within the Rhodobacterales, the *Roseobacter* genera were enriched on the kelp surface under future conditions. One species, *Dinorosebacter shibae DFL12*, synthesizes B_1_ and B_12_ vitamins for the algae host [67]. We suggest that in future conditions, a novel symbiotic relationship where *Planctomycetales* and *Rhodobacterales* are able to thrive on the kelp surface utilizing *M*. *pyrifera* sulfated polysaccharides as an energy source, while resisting *M*. *pyrifera* induced antimicrobial barrage and outcompeting deleterious Flavobacteriales that were enriched in the increased temperature treatment. In the elevated CO2 tanks, there was an increase of carbon monoxide dehydrogenase, which is a bidirectional catalyst of carbon dioxide, which suggests the response of the kelp microbiome to presence of increased CO_2._

Kelp forests located in lower latitudes, like southern California, where upper temperature tolerances (above 18 ^o^C) are reached three to four months out of the year, may be at risk for shorter life cycles, increased seasonal senescence, and increased disease prevalence in an ocean with rising sea surface temperatures unless increasing CO_2_ is sufficient to mitigate this effect. Here we demonstrate that the water and kelp surface microbiomes respond differently to climate stressors. Water microbiomes were most influenced by increased *p*CO_2_ conditions and primarily associated with an increase in Rhizobiales. Kelp surface microbiomes on the other hand, dominated by Alteromonadales, were perturbed by increased temperature, corresponding with increased Flavobacterales, alginate metabolism, and kelp degradation. While kelp microbiomes were largely not disturbed by increases in *p*CO_2_, perturbations in the water column microbes due to pCO_2_ could have an impact in kelp communities by changing the available kelp surface colonizers. The results from this initial case study are preliminary observations which need to be followed up with more highly replicated mesocosms or in situ experiments to verify the findings. Alternatively, environmental sampling across seasonal variances of temperature and pCO_2_ could also be valuable for understanding the microbial influence in this kelp forest ecosystem. Future conditions increased kelp growth and carbon monoxide dehydrogenase corresponding with increased Planctomycetales, Rhodobacterales and sulfatase genes. Our study demonstrates that kelp growth, as a result of changing climate conditions, is directly tied to the host microbiome.

## Materials and methods

*Macrocystis pyrifera* fronds were collected from 72 randomly selected sporophytes from the Point Loma kelp forest, San Diego, California, USA (32°39'59.56"N, 117°14'50.62"W) at 12–15 m deep in August 2012. Fronds containing meristems (shoots or stems) and laminae (blades) were immediately placed in a cooler filled with seawater and transported to San Diego State Coastal Marine Institute Laboratory Point Loma, CA. *M*. *pyrifera* was collected in accordance with U.S. Fisheries and Wildlife Service Permit #SC-13075. At the lab the fronds were trimmed to 7 g and placed in one of four 90 L acrylic incubation mesocosms (n = 18 fronds per mesocosm), each containing recirculating seawater collected from the kelp forest. For four weeks, temperature and *p*CO_2_ levels in each mesocosm were maintained at the following conditions: “present day” at 12 ^o^C and 500 *p*CO_2_, “elevated temperature” at 15 ^o^C and 500 ppm *p*CO_2_, “elevated *p*CO_2_” at 12 ^o^C and 1300 ppm *p*CO_2_, and “future” at 15 ^o^C and 1300 ppm *p*CO_2_ to reflect predicted temperature and CO_2_ increases in nearshore temperate marine environments over the next 100 years [17,68]. Four weeks was selected to provide sufficient time for the kelp to grow and the microbes to change. A 12 hour—light/dark cycle was maintained at 15–20 μmol photons m^-2^ s^-1^ irradiance to simulate light levels experienced in the kelp forest while nutrient levels were maintained by supplemental addition of Proline algae fertilizer to ensure nitrate levels remained above 1–2 uM (Brown, 2014). Each of the 18 pseudo-replicate kelp fronds were resampled from the incubation mesocosms each week, and weighed to monitor growth, while the microbiomes on the kelp fronds and in the water column were collected at the end of the experiment. Measurements of *p*CO_2_ were taken on a daily basis.

At the end of four weeks, replicate microbiomes were collected from the seawater and the kelp frond surfaces incubated in each mesocosm. To sample the water column microbiome, 45 liters of seawater were passed through a 100 μm Nitex filter to exclude zooplankton, larger phytoplankton and organic debris, and then concentrated to 500 mL using tangential flow filtration (TFF) (100 kDa pore, GE Healthcare Life Sciences, Pittsburg, PA). The concentrate was passed through three replicate 0.22 μm Sterivex filters to collect the microbial fraction, (including Bacteria, Archaea, and micro-eukaryotes) [69]. Sterivex filters were sealed with parafilm and stored at -20°C until DNA extraction. Microbes were collected from kelp frond surfaces using a closed circuit two-way 50 mL syringe referred to as a ‘supersucker’ [70]. While pressing the close circuit syringe against the kelp fronds, sterile, 0.02 μm filtered seawater was passed over the surface, dislodging surface microbes from the algal surface and re-circulating the microbes laden water back into the syringe. The microbes and water from the kelp was dispensed through a 0.22 μm Sterivex. The sterivex was sealed with parafilm and immediately stored at -20°C for transport back to a -80°C freezer at SDSU. The sampling process was repeated on three kelp fronds per incubation treatment.

The gDNA was extracted from the Sterivex filters using a modified Macherey Nagel column protocol as described previously [37]. To remove RNA from the eluent, samples were incubated at 37 ^o^C for one hour with RNase A and reprocessed through a DNeasy blood extraction kit (Qiagen, Valencia, CA). Samples were quantified using a Qubit (Thermofisher, Waltham, WA). For kelp surface biological replicates, the gDNA content was low for 9 of the 12 samples (less than 100 ng), thus samples from the two lowest gDNA replicates were pooled into one for each mesocosm (elevated temperature, elevated CO2, and future conditions). All three replicates had to be pooled for the present-day mesocosm, to meet requirements for library preparation.

A total of 19 microbiomes, whole genome sequencing, samples from the water column (n = 12) and kelp surfaces (n = 7) of the four incubation mesocosms were prepared into libraries using 100 ng of gDNA (Illumina PCR Nano prep kit) and quantified using the KAPA qPCR (Kapa Biosystems, Wilmington, MA) and Bioanalyzer (Agilent, Santa Clara, CA) on the high sensitivity assays. Nextera was not chosen due to known biases in fragmentation. Libraries were sequenced over two MiSeq runs using the Illumina V3 2 x 300 sequencing kits (S1 Table). All FASTQ files generated were quality filtered using PRINSEQ [71] and MG-RAST [72] to remove artificial replicate [73], while low quality sequence reads were trimmed to contain less than 5 bases of a Q-score ≤ 15 [74]. The metagenome-microbiomes were uploaded to MG-RAST version 3.5 [75]. Annotations of microbial organisms were generated in MG-RAST at the order and genera level using the Representative Hit Classification setting (e-value = 1 x 10−^5^, minimum percent identity cutoff of 65%, and minimum alignment length cutoff of 50 bp) against the M5 non-redundant database. To identify gene functions associated with the microbes, relative abundances of protein encoded gene annotations were generated at the SEED functional level 3 or gene pathways [69], using the Hierarchical Classification setting with cut-off described above. Rarefaction curves for each microbiome were generated based on ORF detection. Species richness of taxonomy was calculated at the genera level, whereby means of water column replicates were compared using One Way ANOVA followed by a Tukey HSD test with significance of P < 0.05. Kelp microbiome species richness was compared qualitatively.

When conducting a homology based approach to obtain relative abundances of organisms or functions of the microbes from DNA sequences, there is lower confidence in the identification of taxa in the rare tail of the distribution. One of the reasons for this is that metagenomics experiments rarely sample all of the microbes in a given sample, thus one will always have to deal with subsampling error. Although methods exist to quantify this including rarefaction curves and Good’s coverage of singletons [76], there can also be other sources of error including contamination events during sample processing [77] and bioinformatic errors caused by sequencing or analysis with incomplete databases [78]. Here, we quantitatively determined the lower cutoff for our analysis of the genera and functions, and apply this cutoff to the downstream analyses. Consequently, the analysis was conducted on 2,693 genera and 1,007 gene functions across the 12 microbiomes from the water column samples. We then applied these cutoff values to the kelp associated microbiomes since we did not have the added replicates for calculating CV values. First, the relative abundances of each 2,693 genera or 1,007 gene functions were calculated (abundance of category) / total reads x 100%) and binned into seven abundance bins (S2 Table). Second, for each genera and gene function that was identified, the mean, standard deviation, and coefficient of variation (CV) were calculated across the three replicates from each water column treatment. Within each bin, the mean coefficient of variation and standard error for each treatment was calculated and plotted (S1 Fig). The graphs show an elbow formation when the mean CV falls below 20% at the relative abundance bin of 0.01 suggesting an appropriate cutoff level. Thus if a microbiome has 1,000,000 reads passing QC, the acceptable minimum reads of a genera or function category would be 100 reads (total hits/100 X correction factor or 1,000,000 /100 x 0.01 = 100 hits). The calculation was applied and truncated the genera and gene function categories across all microbiomes to 361 genera and 718 gene functions. The same cut-off was applied to the kelp microbiomes.

To identify whether perturbations in abiotic climate conditions affected the structure of the microbiomes, the relative abundances of reads in the 361 genera and 718 gene functions were square root transformed and compared among treatments using hierarchical cluster analyses and non-metric multidimensional scaling (nMDS) based on Euclidean distances in the PRIMER V6 software (PRIMER-E Ltd., Devon, UK) [79]. The experiment had two microbiome environments, water or kelp surface, and each of these were exposed to the four environmental, abiotic conditions: present-day, elevated temperature, elevated *p*CO_2_, and future conditions. To determine if these factors independently or synergistically influenced the microbiomes, the genera and gene function read abundances were independently compared using a two factor permutational multivariate analysis of variance (PERMANOVA) based on Euclidean Distance similarities [80,81] and the relative importance of each factor was determined by calculating their magnitude of effects using variance components analyses (Graham and Edwards, 2001).

To evaluate whether abiotic factors altered the microbiomes in the water and kelp surface environments at a finer scale, microbiomes were each compared to the present-day condition using a SIMPER analysis [79]. The SIMPER analyses were used to quantitatively determine how dissimilar treatment microbiomes were to the present-day control. In addition, the SIMPER analysis was used to determine the relative importance of each of the 361 genera and 718 gene functions to the overall microbiome group dissimilarity between the environmental types and abiotic stressors. All level 3 gene functions from the Cluster Based Subsystem reported from the SIMPER analysis were described based on the more specific gene function ontological classification (level 4). To describe the effects of a treatment (elevated temperature, CO2, or combined) within an environment (water or kelp), genera and gene function feature tables were compared to present-day condition using STAMP [82]. Pairwise comparisons were made using the Two-sided, white’s non parametric t-test [83], with confidence intervals of 95% and a 5% FDR with Benjamini-Hochberg multiple test correction [84].

## Supporting information

S1 Fig Setting an appropriate cutoff based on variability of rare features. The coefficient of variance as a function of relative abundances of (a) genera and (b) gene function Level 3 categories in three biological replicates across four environmental conditions (present-day ○, elevated temperature □, elevated *p*CO_2_ △, and future ◇) indicates a minimum cutoff of 0.01% relative abundance.
(TIFF)

S2 Fig Sequencing depth by sample type. Rarefaction curve of (a) water column and (b) kelp surface microbiomes across the four environmental conditions (present-day ‘black’ ○, elevated temperature ‘red’ □, elevated *p*CO_2_ ‘green’△, and future ‘purple’ ◇. Diversity index was calculated based off of open reading frames, protein annotations from MG-RAST.
(TIFF)

S3 Fig Microbial taxa associated with the water column and kelp surface at the order level from a six month *in situ* sampling in the Point Loma kelp forest. (TIF)

S4 Fig Microbial taxa associated with the water column and kelp surface at the genera level from a six month *in situ* sampling in the Point Loma kelp forest. (TIF)

S1 Table Sequencing summary of water column and kelp surface microbiomes. (DOCX)

S2 Table The proportion of reads that went into each bin for genus and function. (DOCX)
