# Supplementary figures and images for: Elevated temperature drives kelp microbiome dysbiosis, while elevated carbon dioxide induces water microbiome disruption
Source: PLoS One. 2018 Feb 23;13(2):e0192772. doi: 10.1371/journal.pone.0192772 (PMC5825054; doi:10.1371/journal.pone.0192772)

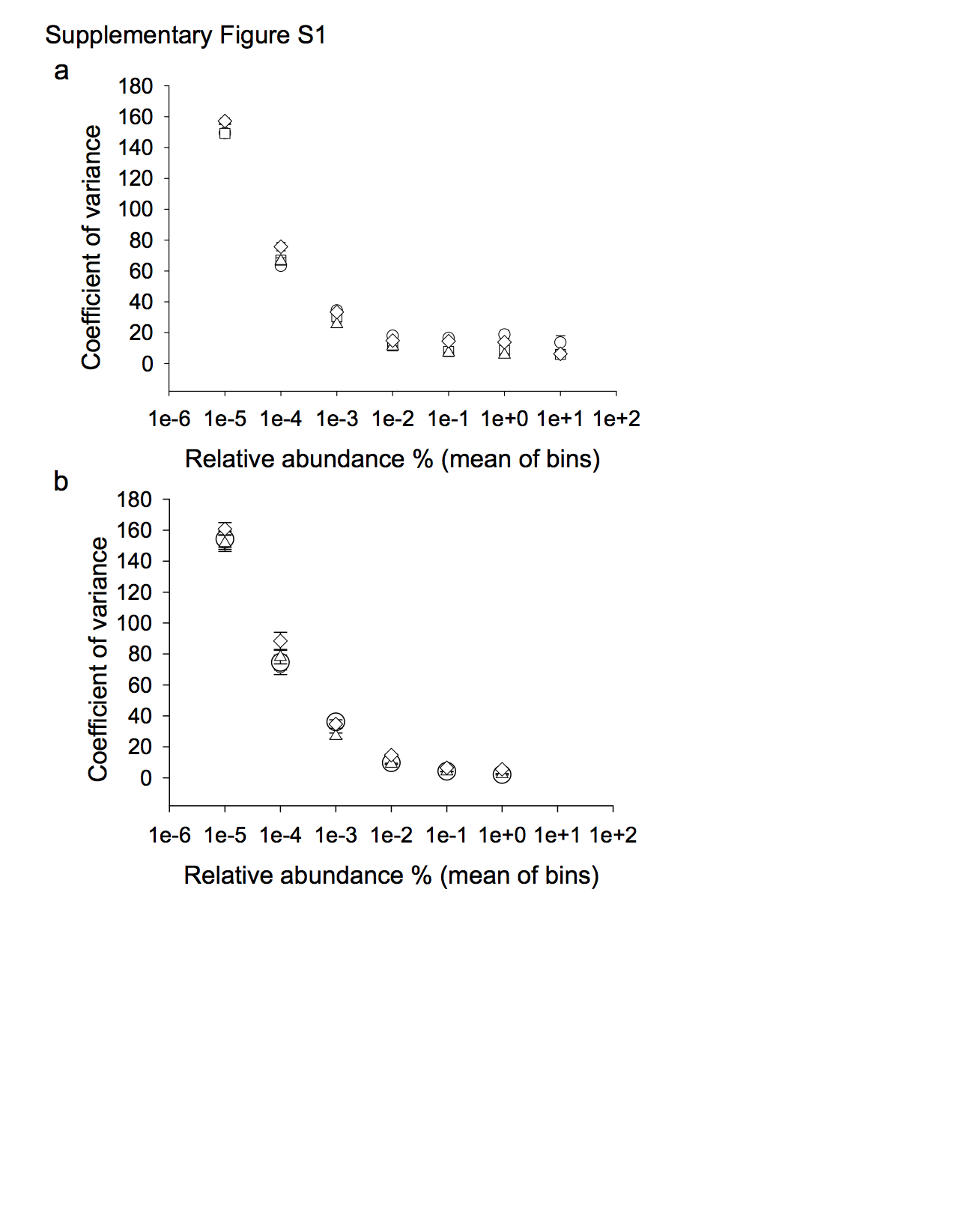

Supplement: S1 Fig — The coefficient of variance as a function of relative abundances of (a) genera and (b) gene function Level 3 categories in three biological replicates across four environmental conditions (present-day ○, elevated temperature □, elevated pCO2 △, and future ◇) indicates a minimum cutoff of 0.01% relative abundance. (TIFF) [file pone.0192772.s001.tiff]

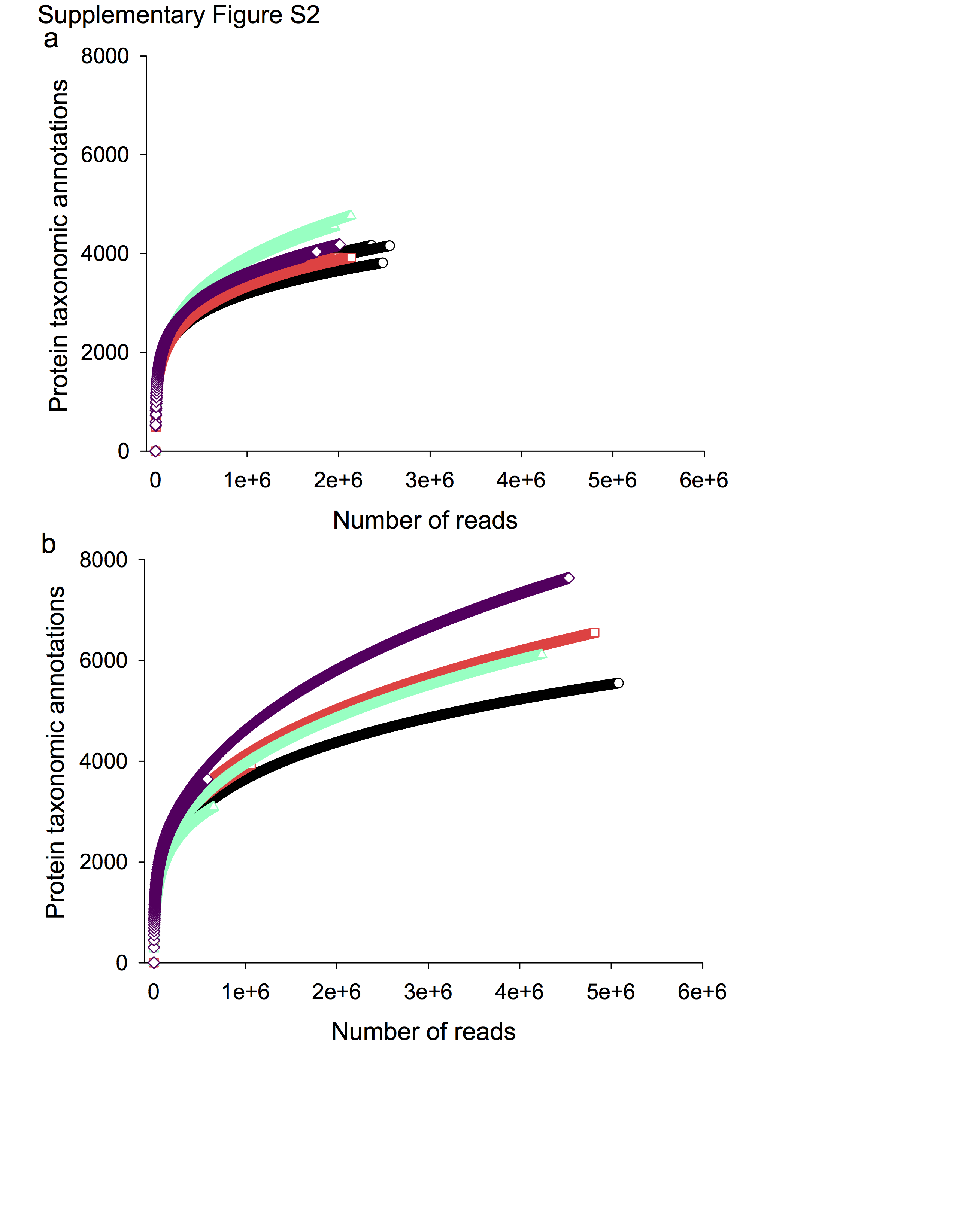

Supplement: S2 Fig — Rarefaction curve of (a) water column and (b) kelp surface microbiomes across the four environmental conditions (present-day ‘black’ ○, elevated temperature ‘red’ □, elevated pCO2 ‘green’△, and future ‘purple’ ◇. Diversity index was calculated based off of open reading frames, protein annotations from MG-RAST. (TIFF) [file pone.0192772.s002.tiff]
