# Supplementary material for: Elevated temperature drives kelp microbiome dysbiosis, while elevated carbon dioxide induces water microbiome disruption
Source: PLoS One. 2018 Feb 23;13(2):e0192772. doi: 10.1371/journal.pone.0192772 (PMC5825054; doi:10.1371/journal.pone.0192772)
Supplement: S1 Table — (DOCX) [file pone.0192772.s005.docx]

Supplementary Table S1: Sequencing summary of water column and kelp surface metagenomes

| Tank Condition | Temp. | *p*CO2 (ppm) | EVNO | MG-RAST ID | Mbp | Reads Pass QC (M) | Reads Pass QC % | Mean RL (bp) | Prot. Ann. (M) |
| --- | --- | --- | --- | --- | --- | --- | --- | --- | --- |
| Present-day | 12 | 500 | W | [mgs631438](http://metagenomics.anl.gov/mgmain.html?mgpage=sample&sample=mgs631438) | 825 | 2.36 | 93 | 322 | 1.00 |
| Present-day | 12 | 500 | W | [mgs631441](http://metagenomics.anl.gov/mgmain.html?mgpage=sample&sample=mgs631441) | 834 | 2.48 | 93 | 314 | 0.53 |
| Present-day | 12 | 500 | W | [mgs631444](http://metagenomics.anl.gov/mgmain.html?mgpage=sample&sample=mgs631444) | 873 | 2.56 | 94 | 320 | 0.60 |
| + temperature | 15 | 500 | W | [mgs631447](http://metagenomics.anl.gov/mgmain.html?mgpage=sample&sample=mgs631447) | 648 | 2.00 | 95 | 301 | 0.54 |
| + temperature | 15 | 500 | W | [mgs631450](http://metagenomics.anl.gov/mgmain.html?mgpage=sample&sample=mgs631450) | 520 | 2.10 | 96 | 233 | 0.51 |
| + temperature | 15 | 500 | W | [mgs631453](http://metagenomics.anl.gov/mgmain.html?mgpage=sample&sample=mgs631453) | 595 | 2.14 | 96 | 261 | 0.55 |
| + *p*CO2 | 12 | 1300 | W | [mgs631456](http://metagenomics.anl.gov/mgmain.html?mgpage=sample&sample=mgs631456) | 657 | 1.96 | 96 | 313 | 0.68 |
| + *p*CO2 | 12 | 1300 | W | [mgs631459](http://metagenomics.anl.gov/mgmain.html?mgpage=sample&sample=mgs631459) | 541 | 1.94 | 97 | 262 | 0.59 |
| + *p*CO2 | 12 | 1300 | W | [mgs631462](http://metagenomics.anl.gov/mgmain.html?mgpage=sample&sample=mgs631462) | 706 | 2.13 | 96 | 309 | 0.75 |
| Future | 15 | 1300 | W | [mgs631465](http://metagenomics.anl.gov/mgmain.html?mgpage=sample&sample=mgs631465) | 638 | 2.01 | 95 | 298 | 0.51 |
| Future | 15 | 1300 | W | [mgs631468](http://metagenomics.anl.gov/mgmain.html?mgpage=sample&sample=mgs631468) | 426 | 1.63 | 96 | 245 | 0.42 |
| Future | 15 | 1300 | W | [mgs631471](http://metagenomics.anl.gov/mgmain.html?mgpage=sample&sample=mgs631471) | 494 | 1.76 | 96 | 266 | 0.42 |
| Present-day | 12 | 500 | KS | [mgs631417](http://metagenomics.anl.gov/mgmain.html?mgpage=sample&sample=mgs631417) | 1661 | 5.08 | 94 | 313 | 3.02 |
| + temperature | 15 | 500 | KS | [mgs631420](http://metagenomics.anl.gov/mgmain.html?mgpage=sample&sample=mgs631420) | 1661 | 4.82 | 96 | 331 | 2.74 |
| + temperature | 15 | 500 | KS | [mgs631423](http://metagenomics.anl.gov/mgmain.html?mgpage=sample&sample=mgs631423) | 367 | 1.06 | 98 | 338 | 0.62 |
| + *p*CO2 | 12 | 1300 | KS | [mgs631429](http://metagenomics.anl.gov/mgmain.html?mgpage=sample&sample=mgs631429) | 1196 | 4.24 | 97 | 271 | 2.64 |
| + *p*CO2 | 12 | 1300 | KS | [mgs631426](http://metagenomics.anl.gov/mgmain.html?mgpage=sample&sample=mgs631426) | 222 | 0.62 | 95 | 312 | 0.31 |
| Future | 15 | 1300 | KS | [mgs631435](http://metagenomics.anl.gov/mgmain.html?mgpage=sample&sample=mgs631435) | 1662 | 4.53 | 95 | 342 | 2.25 |
| Future | 15 | 1300 | KS | [mgs631432](http://metagenomics.anl.gov/mgmain.html?mgpage=sample&sample=mgs631432) | 197 | 0.56 | 96 | 309 | 0.28 |

Temp-temperature Celcius; ENVO-environment; W-water; KS-kelp surface; M-million

Mean RL (bp) - mean read length in base pair

Prot. Ann. (M)- protein annotations x million
