# Supplementary material for: Elevated temperature drives kelp microbiome dysbiosis, while elevated carbon dioxide induces water microbiome disruption
Source: PLoS One. 2018 Feb 23;13(2):e0192772. doi: 10.1371/journal.pone.0192772 (PMC5825054; doi:10.1371/journal.pone.0192772)
Supplement: S2 Table — (DOCX) [file pone.0192772.s006.docx]

Supplementary Table S2 The proportion of reads that went into each bin for each genus and function

| Bin | Lower proportion of reads | Higher proportion of reads |
| --- | --- | --- |
| 0.000001 | 0.0000005 | 0.000005 |
| 0.00001 | 0.000005 | 0.00005 |
| 0.0001 | 0.00005 | 0.0005 |
| 0.001 | 0.0005 | 0.005 |
| 0.01 | 0.005 | 0.05 |
| 0.1 | 0.05 | 0.5 |
| 1 | 0.5 | 5 |
| 10 | 5 | 50 |
